# Induction of MASH in three-dimensional bioprinted human liver tissue

**DOI:** 10.1371/journal.pone.0312615

**Published:** 2024-12-30

**Authors:** Vaidehi Joshi, Dwayne Carter, Alice E. Chen, Keith Murphy, J. William Higgins, Mediha Gurel, Daisy Chilin Fuentes, Sara Brin Rosenthal, Kathleen M. Fisch, Tatiana Kisseleva, David A. Brenner

**Affiliations:** 1 Organovo, Inc., San Diego, California, United States of America; 2 University of California, San Diego, La Jolla, California, United States of America; 3 Sanford Burnham Prebys Medical Discovery Institute, La Jolla, California, United States of America; Medizinische Fakultat der RWTH Aachen, GERMANY

## Abstract

Metabolic dysfunction-associated steatohepatitis (MASH), formerly known as nonalcoholic steatohepatitis (MASH), is a major risk factor for cirrhosis and hepatocellular carcinoma (HCC) and a leading cause of liver transplantation. MASH is caused by an accumulation of toxic fat molecules in the hepatocyte which leads to inflammation and fibrosis. Inadequate human “MASH in a dish” models have limited our advances in understanding MASH pathogenesis and in drug discovery. This study uses complex multicellular 3D bioprinting, combining hepatocytes with nonparenchymal cells in physiologically relevant cell ratios using biocompatible hydrogels to generate bioinks Bioprinted human liver tissues consisting of the four major cell types, (hepatocytes, liver endothelial cells, Kupffer cells, and hepatic stellate cells) are generated from cells purified from normal human livers, using this complex bioprinting platform. These liver tissues are incubated in a cocktail consisting of fatty acids, lipopolysaccharide (LPS), and fructose to produce a MASH phenotype in comparison to liver tissues incubated in control media. Furthermore, these bioprinted liver tissues are of sufficient size to undergo histological processing and immunohistchemistry comparable to classic clinical pathological analysis. The MASH liver tissues develop hepatocyte steatosis, inflammation, and fibrosis, in response to the MASH induction media. Additionally, the transcriptome of the MASH tissues differed significantly from the healthy tissues and more closely resembled the transcriptome of biopsies of MASH livers from patients Thus, this study has developed a MASH bioprinted liver tissue suitable for studies on pathophysiology and drug discovery.

## Introduction

Metabolic dysfunction-associated steatohepatitis (MASH), formerly known as nonalcoholic steatohepatitis (NASH), is a major risk factor for cirrhosis and hepatocellular carcinoma (HCC) and a leading cause of liver transplants [1]. MASH is caused by an accumulation of toxic fat molecules in the hepatocyte which over time leads to inflammation and tissue damage. As the liver begins to accumulate scar tissue, a process called fibrosis, its ability to function declines. The fibrosis grade in MASH is directly associated with liver mortality and total mortality in patients. Therefore, the fibrosis score is the key intermediate biomarker for prognosis in patients and in preclinical models of MASH.

The Food and Drug Administration (FDA) Modernization Act 2.0 “allows for alternatives to animal testing for purposes of drug and biological product applications.” In response, human *in vitro* liver models are being developed to generate cultured cell models of liver diseases. A particular emphasis is the goal to develop “MASH in a dish” as a novel approach to test the effectiveness of anti-MASH drugs. Initial studies used 2D cultures of primary human liver cells. However, expression analysis of primary human hepatocyte cultures demonstrated dramatic dedifferentiation of hepatocytes starting at 30 minutes after plating and continuing with 5000 genes being differentially expressed after 4 hours. Expression of some key hepatocyte enzymes decreased by 90%. Thus, alternative approaches were needed to be developed to use human liver cells in drug development [2, 3]. The technological approaches include precision cut liver slices, human liver spheroids, human liver organoids, bioprinted human liver tissues, and microphysiological systems. The FDA Modernization Act 2.0 specifically contemplates these approaches, calling for “human biology-based methods, such as cell-based assays, microphysiological systems, or bioprinted or computer models.”

Complex multicellular bioprinting which focuses on combining hepatocytes with nonparenchymal cells in physiologically relevant cell ratio involves use of biocompatible hydrogels to generate bioinks [4]. Bioprinted human liver tissues can be assembled with a 3D printer, such as the ExVive Human Liver Tissue developed with NovoGen Bioprinter [5]. This represents a substantial innovation in the study of progressive liver injury, as it addresses many of the shortcomings associated with traditional 2D *in vitro* culture models, including longevity, multicellular complexity, spatial organization, and architectural control. Bioprinted human liver tissues are comprised of primary hepatocytes, hepatic stellate cells (HSCs), Kupffer cells (KCs), and liver endothelial cells arranged in precise compartments within a three-dimensional tissue structure [6]. Macroscopically, the tissues resemble a clinical biopsy, with sufficient size to enable biochemical, transcriptional, and histological assessment from one bioprinted culture well. There are two basic approaches to developing “MASH in a dish” from 3D bioprinted liver tissues. As previously described [7], one approach is to print with cells from patients with MASH and culturing the tissues in standard media. This approach depends on the persistence of genetic and epigenetic changes in the hepatic cells to develop a MASH phenotype. Our study describes a second approach, in which hepatic cells from normal livers are bioprinted, and then the bioprinted liver tissue is incubated in a MASH cocktail to induce MASH over time.

## Methods

### Bioprinting 3D liver tissues

3D liver tissues comprising of cryopreserved primary human hepatocytes (Gibco, Thermo Fisher Scientific), hepatic stellate cells, liver endothelial cells, and Kupffer cells, were fabricated by Organovo, Inc. (San Diego, CA, USA) with a NovoGen Bioprinter onto 24-well transwell culture inserts using patented protocols as previously described [8–13]. Briefly, stellate cells and liver endothelial cells were expanded to desired numbers using standard cell cultures techniques, for tissue fabrication. On the day of print, stellate and liver endothelial cell suspensions were first mixed (10–30% stellate cells: 90–70% endothelial cells) and then combined with a hydrogel to form the non-parenchymal cell bioink (150e6 cells/mL formulated in a thermo-responsive hydrogel). Cryopreserved hepatocytes and kupffer cells were then thawed and prepared for use per the manufacturer’s instructions. 100% cellular bioink consisting of hepatocytes and Kupffer cells (70–95% hepatocytes: 30–5% kupffer cells) was generated via compaction. Both bioinks were then drawn-up in two separate syringes and loaded onto separate heads of the Novogen Bioprinter. A computer script pre-designed for the desired geometry was then executed to deposit the bioinks in a two-compartment geometry. The tissues were printed onto the membranes of standard 24-well 0.4 μm transwell culture inserts (Corning) via continuous extrusion of the bioinks, with the non-parenchymal cells forming the border and the hepatocyte paste with Kupffer cells forming the fills in each compartment. Following fabrication, all the tissues were fed daily with 600 μL of appropriate liver culture media and incubated at 37°C under humidified atmospheric conditions supplemented with 5% CO2.

### Steatosis/MAFLD induction in bioprinted tissues with glucose, fructose and fatty acids

Following fabrication, tissues were cultured overnight at 37°C and 5% CO2 in plating media, consisting of DMEM no glucose, supplemented with primary hepatocyte plating supplements with FBS (CM 3000, Life Technologies, Carlsbad, CA), EGM-2 (Lonza, Basel, Switzerland), and glucose solution (Sigma Aldrich, St. Louis, MO) at a final concentration of 11 mM. 12–18 hours post-fabrication the tissues were transitioned to 3D bioprinted liver tissue maintenance media, comprised of the DMEM no glucose base with 11 mM glucose, and primary hepatocyte maintenance supplements (CM 4000, Life Technologies; Carlsbad, CA), and EGM-2. Tissues were matured over 3 days post-fabrication then qualified and utilized for experimentation. Tissues designated for steatosis induction were cultured in media comprised of the same components as the maintenance media above + fructose (Sigma Aldrich, St. Louis, MO), palmitic acid and oleic acid (Sigma Aldrich, St. Louis, MO) formulated in 10% BSA solution (Sigma Aldrich, St. Louis, MO) for 21 days.

### Hepatocyte donor-dependent response to MAFLD and MASH induction

3D liver tissues were fabricated as described in the methods above. To assess the impact of steatosis and MASH stimulants on different hepatocyte donors, separate parenchymal bioink syringes were prepared using four distinct hepatocyte donors. Non-parenchymal cell bioink was held constant.

### Drug efficacy testing with GS0976 treatment on 3D bioprinted tissues

To Assess drug efficacy, 3D bioprinted liver tissues were treated with leading MASH clinical candidate, acetyl CoA carboxylase (ACC) inhibitor GS-0976 at 0.37 μM, 1.1 μM, 3.3 μM, and 10 μM. Media was changed daily for up to 21 days with fresh compound added daily.

### Optimized MASH induction utilizing fatty acid treatment along with bacterial endotoxin LPS for Bulk RNA-Seq analysis

MASH phenotype was induced by adding unsaturated fatty acid–Oleic acid (Stock:150mM in 0.1 N NAOH), saturated fatty acid–Palmitic acid (Stock:100mM in 0.1 N NAOH) and bacterial endotoxin–LPS in 10% BSA to the liver tissue maintenance media consisting of Williams E hepatocyte media with maintenance supplements (CM 4000, Life Technologies, Carlsbad, CA) and EGM-2 (Lonza, Basel, Switzerland), for a final fatty acid concentration of 3mM and LPS concentration of 10 μg/mL. Tissues were allowed to mature in culture for three days following fabrication prior to initiation of the experiment. All tissues were substantially free of the hydrogel at the start of the study. On day 3 post print and day 0 of treatment, tissues were divided into treatment (Tx) and control groups. For MASH induction in Tx group, tissues were fed with MASH induction media, described above, daily through the course of the study. All tissues were fed daily for a total of 21 days post-fabrication. Feeding volumes were maintained at 600 μL in the bottom chamber and 200 μL in the top chamber. Tissues were then processed for histology and bulk RNA seq.

### RNA isolation and Bulk RNA-Seq

Prior to RNA extraction, media was removed and bioprinted liver tissues were frozen in liquid nitrogen and stored at −80°C until ready for RNA extraction. Tissues were homogenized using a homogenizer, and total RNA was isolated from homogenized tissue with RNeasy Mini Kit (QIAGEN, Valencia, CA). Strand-specific mRNA-sequencing libraries (polyA+) were generated and barcoded using Illumina’s TruSeq stranded mRNA library prep kits. Each RNA-Seq library was sequenced on an Illumina HISEQ.

### Bulk RNA-Seq analysis

Quality control of the raw fastq files was performed using the software tool FastQC https://paperpile.com/c/nzZpyN/ANfe [14] v0.11.8. Sequencing reads were trimmed with Trimmomatic [15] v0.38 and aligned to the human genome (GRCh38.p13 [16]) using the STAR aligner [17] v2.7.10b_alpha_230301. Read quantification was performed with RSEM v1.3.0 [18] and the Gencode release 43 annotation [19]. The R BioConductor packages edgeR [20] and limma [21] were used to implement the limma-voom [22] method for differential expression analysis. In brief, lowly expressed genes—those not having counts per million (cpm) ≥ 1in at least 2 of the samples—were filtered out and then trimmed mean of M-values (TMM)^1^ [23] normalization was applied. The experimental design was modeled upon condition and batch (~0 + condition + batch). The voom method was employed to model the mean-variance relationship in the log-cpm after which lmFit was used to fit per-gene linear models and empirical Bayes moderation was applied with the eBayes function. Significance was defined by using an adjusted p-value cut-off of 0.05 after multiple testing correction [24] using a moderated t-statistic in limma and a minimum absolute log-fold-change threshold of 2. Functional enrichment of the differentially expressed genes was performed using ToppGene [25] using the GO, KEGG, REACTOME, and DisGesNet databases.

### Histological processing of paraffin embedded tissues

On Day 7, 14 and 21 of treatment, liver tissues were fixed in 5% formalin for 48 hours at 4°C. After 48 hours, the fixation solution was removed and replaced with 70% ethanol. Tissues with the attached transwell membrane were submerged in 60°C liquid Histogel (American MasterTech Scientific) in a plastic biopsy mold and allowed to solidify on a cold plate. Histogel blocks with embedded liver tissues were then processed for paraffin embedding using a tissue processor. Following infiltration with paraffin, liver tissues were embedded in paraffin molds and 4 μm cross- sections were cut with an automated rotary microtome. Tissues were then stained using Trichrome One-Step Blue & Red stain according to the manufacturer’s protocol (American MasterTech Scientific). Stained and cover-slipped slides were then imaged using ZEISS Axio Scan.Z1 automated slide scanner.

### Statistical analyses

Statically analyses were conducted samples sizes of n ≥ 3. Statistical significance was defined as p < 0.05. Values reported as means ± SD. Analyses were performed with Graphpad Prism statistics program for Microsoft Windows, Version 7.0. (GraphPad Software, La Jolla, CA).

## Results

### Development of a 3D bioprinted MASH model

#### Modeling MAFLD in response to treatment with high sugars and fatty acid in human 3D bioprinted liver tissue

**Fig 1:**
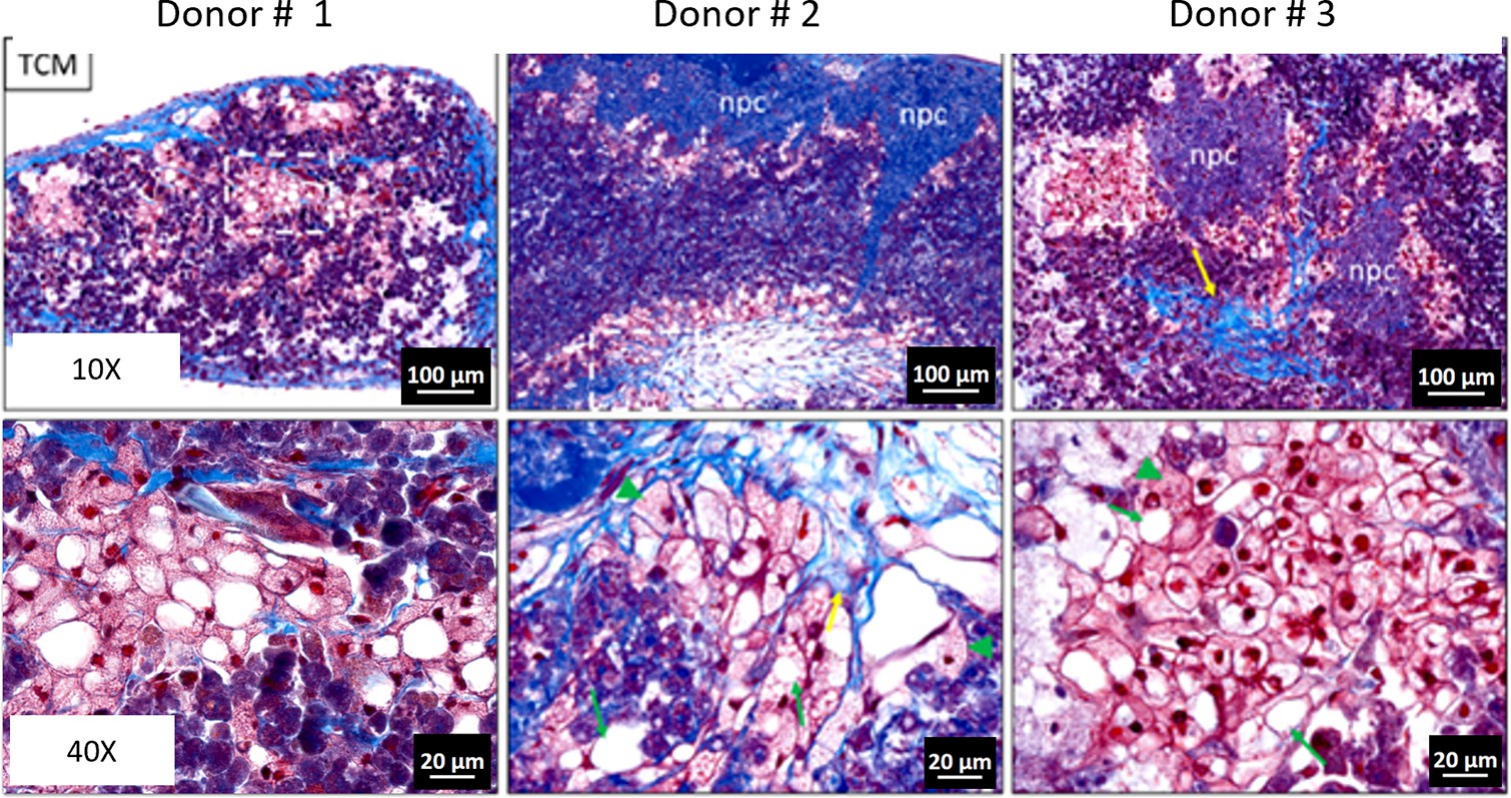
MAFLD like phenotypes can be induced using G/F/PA treatment. Trichrome staining of sections of tissues treated with G/F/PA for 21 days. Top, 10x; Bottom, magnified inset 40x of dotted white box. Collagen fibrils (black arrow); putative macrovesicular (M) and microvesicular (m) steatosis, putative ballooning hepatocytes (B). npc, non-parenchymal cells.

The progression of MAFLD to MASH involves the excessive accumulation of fats in hepatocytes, known as steatosis, followed by inflammation, oxidative stress, hepatocellular injury, and eventually fibrosis. To study MAFLD, 3D bioprinted liver tissues were fabricated as described above, using primary liver cells from healthy human livers, followed by treatment with a combination of glucose (G), fructose (F), and saturated (palmitic acid (PA)) and unsaturated (oleic acid (OA)) fatty acids. In response to these stimuli, the tissues exhibited steatosis (S1 Fig), increased inflammatory cytokine release (S2 Fig), deposition of extracellular matrix and hepatocellular ballooning (S3 Fig), and stellate activation (S4 Fig), consistent with hepatocellular injury and fibrosis. Following up on this observation, we extended testing across multiple hepatocyte donor lots to explore both protocol reproducibility and donor response. We found that MASH-like phenotypes could be induced across multiple hepatocyte donors (Fig 1), with range in response. For example, similar to Donor # 1, Donor # 2 and # 3 exhibited large and small droplet lipid accumulation in cells with evidence of hepatocyte degeneration, putative ballooning, and fibrosis, highlighting protocol reproducibility across tissues of different hepatocyte donor composition. While we observed key hallmarks of the disease in all donors, the severity between donors was variable. In all cases, non-parenchymal cell donors were held constant.

#### Supplementing MAFLD induction media with LPS can result in complex MASH phenotypes across multiple hepatocyte donors

**Fig 2:**
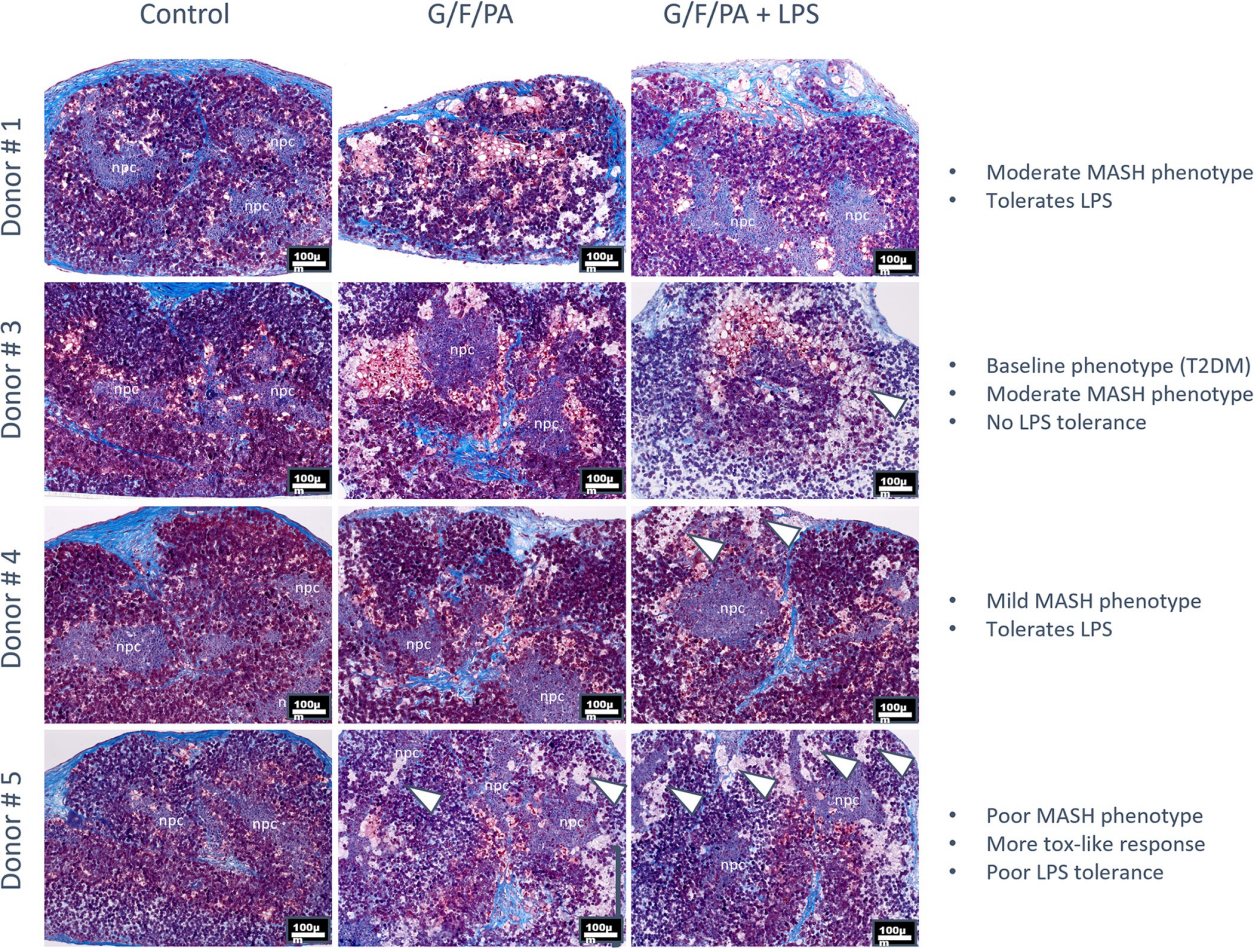
Heterogeneity of hepatocyte donor response. Trichrome staining of sections of tissues treated with G/F/PA or G/F/PA/LPS for 21 days (10x). Uninduced tissues included as Controls. Degenerating or ballooning cells (white arrowhead). Dissociated cells (black bracket). npc, non-parenchymal cells. T2DM, Type 2 Diabetes Mellitus.

Beyond the role that high sugars and fats play in the pathogenesis of MAFLD progression, the overconsumption of nutrients can also compromise intestinal epithelial barrier integrity. This in turn can result in increased circulating levels of the bacterial endotoxin lipopolysaccharide (LPS), a known potent activator of Kupffer cells also shown to play a role in the pathogenesis of the MAFLD and MASH. To drive disease progression in 3D bioprinted tissues containing Kupffer cells, we investigated whether the combined exposure to sugars, fats, and LPS could elevate disease phenotypes to include inflammation and fibrosis, characteristic of MASH in vivo. To evaluate this across a broader range of hepatocyte donors, 3D bioprinted liver tissues were made using hepatocytes from different donors and treated with G/F/PA, with or without LPS. The responses varied among donors (Fig 2). Donor # 3 exhibited a low % of steatosis at baseline in uninduced tissues (likely related to donor condition of Type 2 Diabetes Mellitus), was moderate in MASH phenotypic response to G/F/PA, but exhibited significant toxicity and sensitivity to LPS, as evidenced by severe tissue dissociation. In response to treatment with G/F/PA, hepatocyte donors #4 and #5 exhibited a lower incidence of steatosis and incidence of MASH phenotypes, compared to donors #1 and #3. Additionally, donor #5 showed an increased incidence of putative ballooning, pronounced cell degeneration and tissue dissociation upon treatment with G/F/PA that was further exacerbated to show fibrosis upon LPS exposure, suggestive of poor tolerance and increased sensitivity to insult. These results demonstrate that MASH-like phenotypes can be achieved across multiple hepatocyte donor backgrounds using a consistent induction regimen, albeit with noticeable variability between donors. Nonetheless, the ability to include hepatocytes from diverse donor origins (both normal and diseased) enables in vitro modeling of donor heterogeneity as well as donor characteristic dependent susceptibility to MAFLD and MASH, an aspect of *in vitro* testing currently not available.

#### Application of 3D bioprinted MAFLD liver tissues to drug efficacy screening

A major challenge for pharmaceutical companies continues being the inability to predict clinical outcomes based on efficacy results of drugs from rodent models. To date, nearly all drugs that have shown efficacy in MASH rodent models have failed to demonstrate similar efficacy in patients. Hence, there is a pressing need for predictive human pre-clinical MASH models to reduce the costs associated with failed drug testing in clinical trials. To impact the development of novel treatments for MASH, a new model must both replicate the clinical phenotype and show clinically relevant prevention and reversal with treatment.

Increased de novo lipogenesis (DNL) contributes to the development of steatosis and progression from MAFLD to MASH. Acetyl CoA Carboxylase (ACC) catalyzes the rate-limiting step in DNL, and its inhibition using the potent and selective inhibitor, GS0976, has been shown to reduce hepatic DNL and steatosis in MASH patients [26]. To evaluate the application of the MASH- induced tissue model described in this paper for drug efficacy studies, liver tissues were treated with GS-0976, an acetyl CoA carboxylase (ACC) inhibitor, following a therapeutic approach.

**Fig 3:**
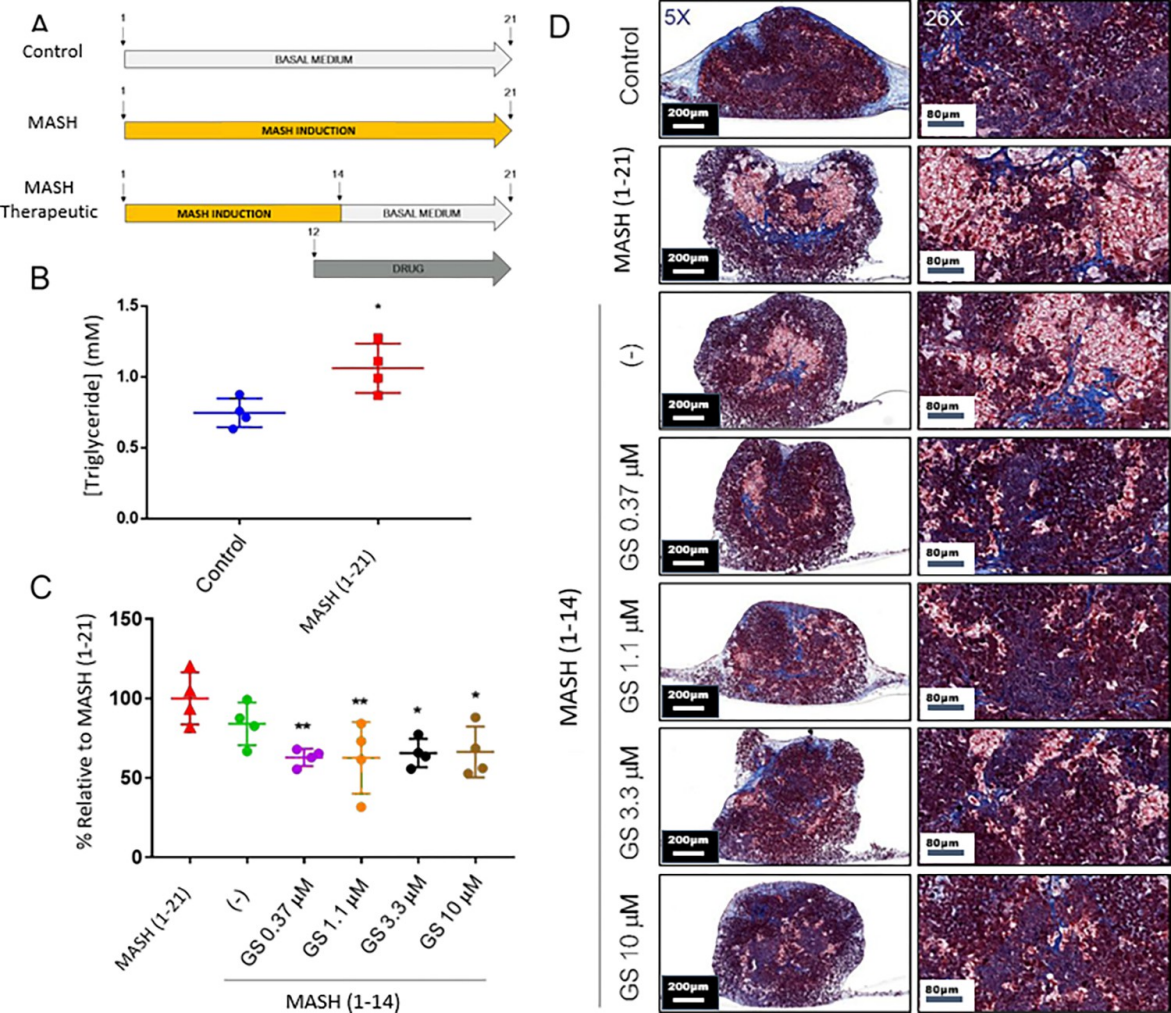
Pilot drug efficacy testing with GS0976. (A) Schematic of therapeutic intervention paradigm. Numbers indicate treatment days. (B) Triglyceride quantification in tissues +/- treatment with MAFLD conditions (G/F/PA) for 21 days. Unpaired T-test; *p<0.05. n = 4 each. (C) Triglyceride quantification in tissues treated with MAFLD +/- GS0976 at increasing concentrations. One-way ANOVA with multiple comparisons; ** p<0.01, *p<0.05. n = 4 each. (D) Trichrome staining of tissue sections from control and MAFLD conditions +/- GS0976 at increasing concentrations for 21 days. 5x and 26x shown.

To assess the effects of GS0976 on MAFLD-associated phenotypes in our system, a therapeutic treatment regimen was tested (Fig 3A). MAFLD induction (G/F/PA) for 21 days led to detectable increase in the quantity of triglycerides (Fig 3B). To mimic patients who undergo dietary restriction following diagnosis, one group of bioprinted liver tissues was treated with MAFLD conditions for 14 days, followed by cessation and continued culture in basal medium for the remaining 7 days. To mimic patients who combine dietary restriction with drug treatment, MAFLD induction was carried out for 14 days, with administration of GS0976 at 0.37, 1.1, 3.3, or 10 μM on days 12–21. Subsequent analysis of lipid content showed that cessation of MAFLD conditions alone (MAFLD 1–14) did not affect triglyceride levels compared to tissues with constitutive induction (MAFLD 1–21), whereas combined treatment with GS0976 led to significant reduction in triglyceride levels at all doses tested (Fig 3C), with no dose-dependency. Consistent with this, histological evaluation revealed reduced incidence of steatosis in tissues co- treated with GS0976 (Fig 3D) with no observable difference in fibrosis.

#### Histological analysis and Bulk RNA-Seq on MASH tissues induced with optimized conditions

**Fig 4:**
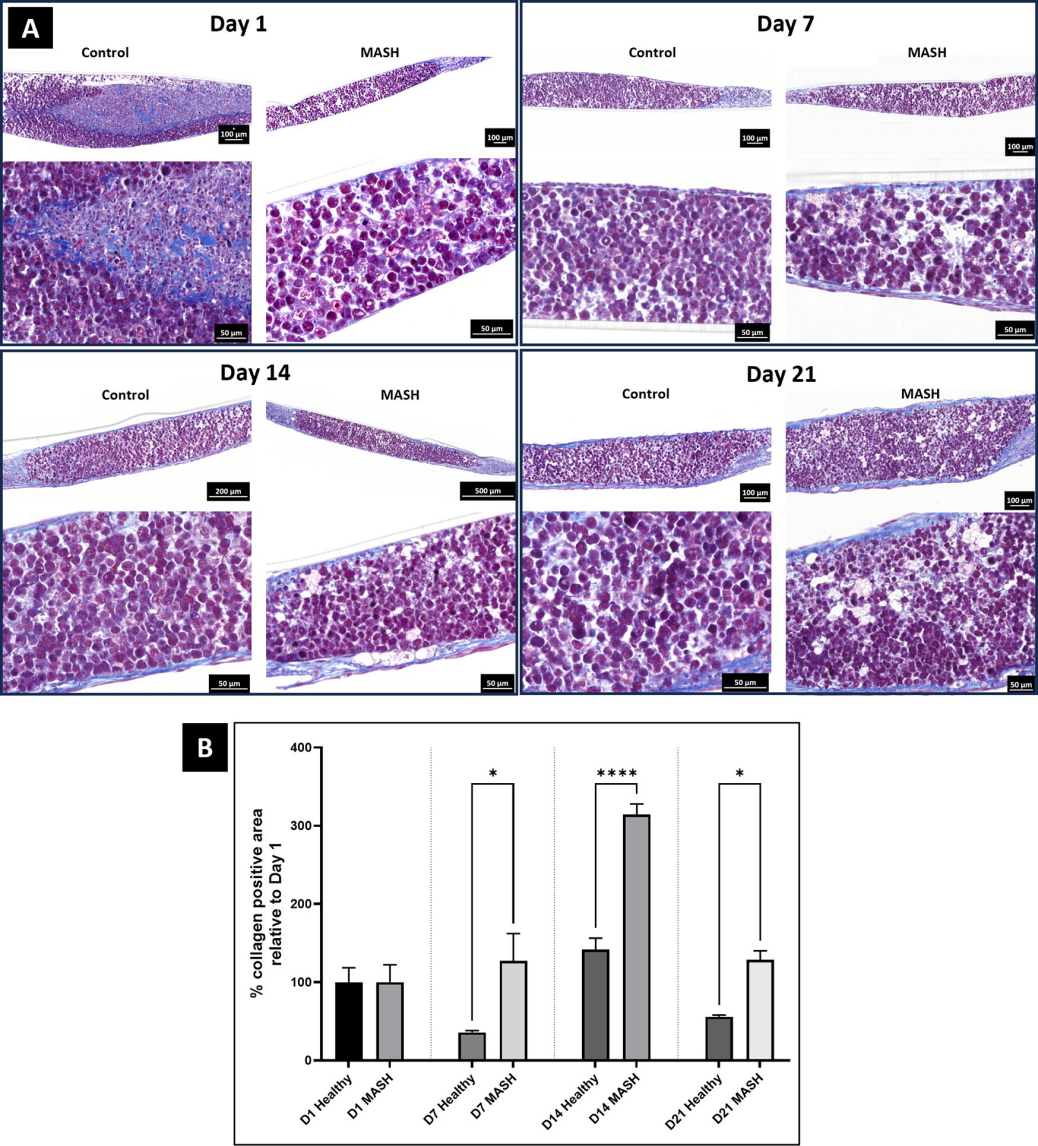
MASH-like phenotype induction at 21 days. (A) Trichrome One-Step Blue & Red stained sections from healthy and MASH-induced bioprinted tissues reveal collagen deposition as represented by the cobalt blue stain in the images above. Also highlighted with black arrows are ballooned hepatocytes. MASH-induced tissues showed increased fibrosis, as seen by the collagen deposition, as well as more ballooned hepatocytes compared to healthy controls. (B) Bar graph represents quantitative measurements of % collagen positive area. 4 sections per tissue (n = 3 tissues) were averaged to get representative quantitative data for each Healthy and MASH-induced condition. Graph above shows data normalized to Day 1 tissues post-treatment. Statistics (One-way ANOVA with multiple comparisons) were calculated with using GraphPad Prism software, * p<0.05, n = 3.

Toward identifying the minimum stimulus to induce MASH-like phenotypes, we next explored eliminating the addition of sugars while still retaining fatty acids and LPS. The bioprint consisted of the four major hepatic cell types and incubated in media consisting of 90% Williams E + 10% EGM (45mL WSE + 5mL ECM). At day 3 post-printing, the cultures were treated with the MASH cocktail containing OA, PA, and LPS. Media and tissue were collected at post-print Day 1, 7, 14, and 21. MASH tissues showed increased steatosis, ballooned hepatocytes, and fibrosis, similar to human MASH livers (Fig 4A). Collagen deposition quantified as the % collagen positive area by Trichrome staining demonstrated increased fibrosis for MASH tissues at all time points (Fig 4B).

#### Transcriptional changes over time and by condition in bioprinted liver tissues

**Fig 5:**
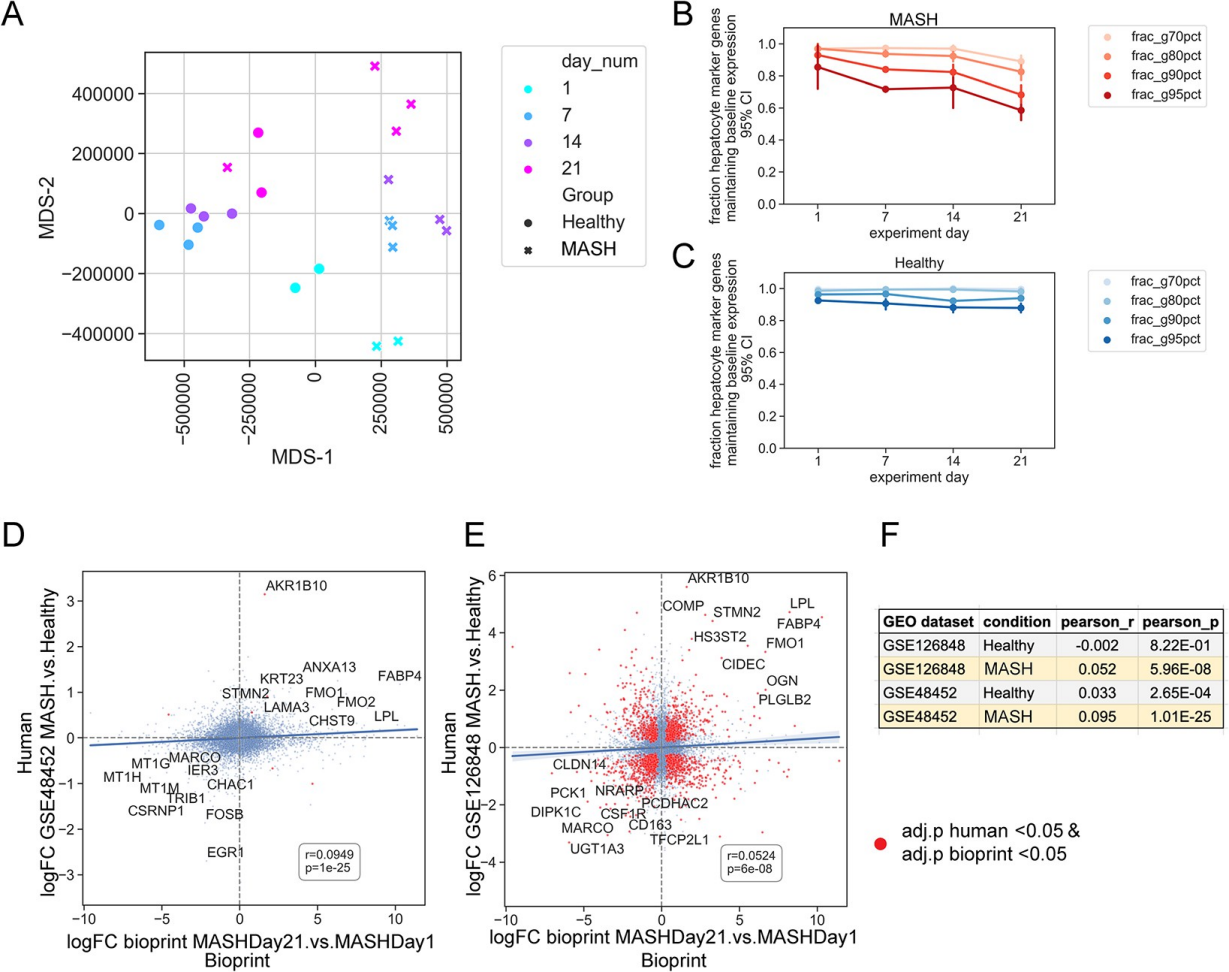
Characterization of bioprinted tissues in relation the human MASH. (A) Multidimensional scaling scatterplot (MDS), computed on batch-corrected, normalized counts. Points are color-coded by experiment day. Healthy bioprinted tissues are indicated by circles, and MASH bioprinted tissues are indicated by x’s. (B) Line plot showing the fraction of hepatocyte marker genes which maintain expression in MASH bioprinted tissues greater than 70%, 80%, 90%, or 95% of baseline expression (indicated by colors). Error bar indicates 95% CI. (C) Line plot showing the fraction of hepatocyte marker genes which maintain expression in Healthy bioprinted tissues greater than 70%, 80%, 90%, or 95% of baseline expression (indicated by colors). Error bar indicates 95% CI. (D) Scatterplot showing the log fold change between MASH day21 bioprinted tissues and MASH day1 bioprinted tissues on the x-axis, and log fold change between human MASH and healthy (GSE48452) on the y-axis. Best-fit regression line with 95% confidence interval shown. Top 10 concurrently upregulated and downregulated genes labeled. Pearson correlation and p-value annotated on graph. Red dots indicate genes which were significantly changed in both bioprinted tissues and native human livers (adj. p <0.05). (E) Scatterplot showing the log fold change between MASH day21 bioprinted tissues and MASH day1 bioprinted tissues on the x-axis, and log fold change between human MASH and healthy on the y-axis (GSE126848). Best-fit regression line with 95% confidence interval shown. Top 10 concurrently upregulated and downregulated genes labeled. Pearson correlation and p-value annotated on graph. Red dots indicate genes which were significantly changed in both bioprinted tissues and human livers (adj. p <0.05). (F) Summary table of both GSE126848 and GSE48452 and how they correlate to MASH and Healthy bioprinted tissues.

Following dimensionality reduction, which was applied to batch-corrected, normalized expression, the human liver bioprints were stratified both by MASH vs. healthy, and by experiment day (Fig 5A). This indicated that bioprints subjected to a MASH cocktail are transcriptionally distinct from their healthy counterparts, which were not incubated in MASH cocktail, and that the bioprints changed their expression patterns over time. (We noted that there was one MASH sample from day 21 which clustered with the healthy samples.)

### Stability of hepatocyte marker genes over time

We examined the expression of 110 canonical markers of hepatocytes (derived from the panglao database) over time in both MASH and healthy bioprinted liver tissues (Fig 5B and 5C). We observed that, in general, expression of hepatocyte marker genes was maintained over time in both MASH and healthy, with a slight falloff of expression in MASH. For example, on MASH day 21, 81% of hepatocyte marker genes maintained expression greater than 80% of the baseline expression observed on MASH day 1 (Fig 5B). This was lower than the 98% of hepatocyte marker genes maintaining expression levels greater than 80% of the baseline expression observed at healthy day 1, but still demonstrated continued widespread expression of the hepatocyte marker genes.

### A comparison of MASH bioprinted tissues to human MASH

We evaluated how much was shared between MASH human bioprinted tissues and two previous studies of transcriptomic differences between human MASH livers and human healthy livers. The datasets were downloaded from GEO (GSE48452; PMID: 23931760, and GSE126848; PMID: 30653341). Due to the batch effects observed in our data, we used the comparison of MASH Day 21 bioprinted tissues to MASH Day 1 bioprinted tissues, instead of comparing to the healthy bioprinted tissues at the respective timepoints. The log fold change between MASH day 21 bioprinted tissues and MASH day 1 bioprinted tissues significantly correlated with the log fold change between human MASH and human healthy livers, in both GSE48452 and GSE126848 (Fig 5D–5F). Some notable genes concurrently upregulated in MASH Day 21 bioprinted tissues and human MASH livers include LAMA3, FABP4, and LPL (Fig 5D and 5E). As a negative control, we compared the log fold change between healthy day 21 bioprinted tissues and healthy day 1 bioprinted tissues to human MASH (Fig 5F). In both GSE48452 and GSE126848, the correlation with human MASH was markedly lower and less significant in Healthy bioprinted tissues, relative to MASH bioprinted tissues.

### Pathway and gene set analysis

**Fig 6:**
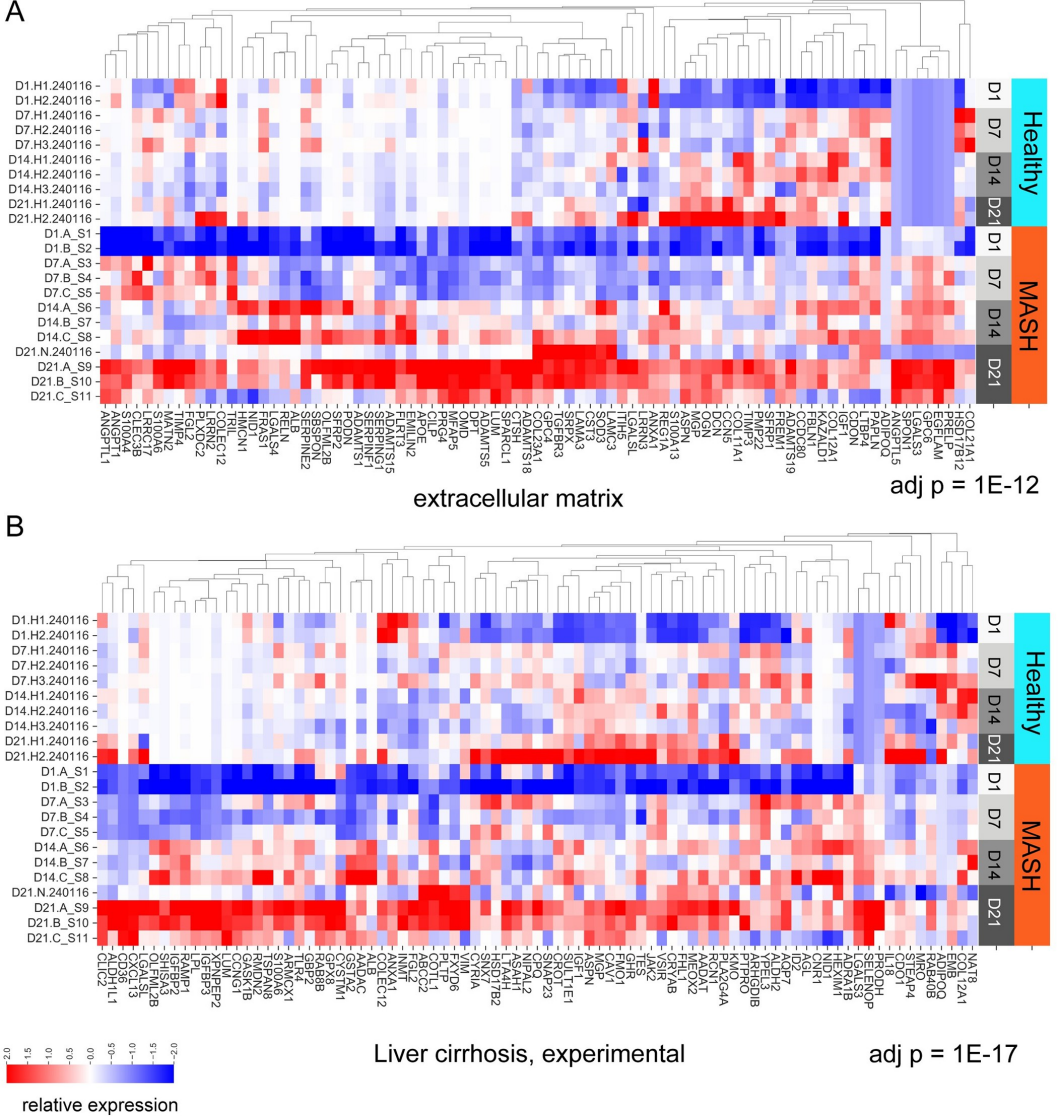
Pathway analysis of bioprinted MASH. (A) Heatmap showing the relative expression of genes which were significantly upregulated in MASH Day 21 bioprinted tissues vs MASH Day 1 bioprinted tissues, and which were found in the gene ontology extracellular matrix pathway. Relative expression is shown across both Healthy and MASH bioprinted tissues, and across all experimental timepoints. The extracellular matrix pathway was significantly enriched for MASH Day 21 bioprinted tissues genes (adj p = 1E-12). (B) Heatmap showing the relative expression of genes which were significantly upregulated in MASH Day 21 bioprinted tissues vs MASH Day 1 bioprinted tissues, and which were found in the liver cirrhosis, experimental gene set, from DisGeNet. Relative expression is shown across both Healthy and MASH bioprinted tissues, and across all experimental timepoints. The liver cirrhosis gene set was significantly enriched for MASH Day 21 bioprinted tissue genes (adj p = 1E-17).

Functional enrichment analysis was conducted on genes significantly upregulated in MASH Day 21 bioprinted tissues compared to MASH Day 1 bioprinted tissues. We identified extracellular matrix (adj p = 1E-12) and liver cirrhosis (adj p = 1E-17) among the significantly enriched pathways (Fig 6A and 6B). Interestingly, some genes are uniquely upregulated by MASH, while other genes show similar expression patterns in healthy bioprinted tissues and MASH bioprinted tissues. (Fig 6A and 6B).

## Discussion

Metabolic dysfunction-associated steatohepatitis (MASH), represents a significant and growing public health concern worldwide, characterized by hepatic steatosis, inflammation, and varying degrees of fibrosis. In vitro models of MASH play a crucial role in elucidating the underlying mechanisms of disease pathogenesis, identifying potential therapeutic targets, and evaluating the efficacy of pharmacological interventions. Several preclinical models of MASH have been developed and are being used extensively to study these areas. While standard cell culture models allow for elucidation of certain molecular pathways involved in MASH pathogenesis, they lack three-dimensional architecture and cellular heterogeneity observed in native liver tissue, potentially limiting their physiological relevance and inability to fully capture the multifactorial nature of the disease. Precision cut liver slices from MASH livers retain the complex multicellular architecture of the liver including hepatocyte arrangement, sinusoidal structure, and cellular interactions, but the metabolism deteriorates over a short period of time (usually reported as 2 days) and they must be prepared from fresh MASH livers Human liver organoids from iPSCs (induced pluripotent stem cells) retain the genetics of the patients but fail to fully differentiate into hepatocytes or have the liver architecture. Human liver spheroids incubated in a MASH cocktail develop several phenotypic characteristics of a MASH liver, but the small spheroid size could preclude extensive histological comparisons to MASH livers. Microphysiological systems (MPS) or “liver on a chip” in which 3D cultures are perfused, maintain many of the physiological functions of the liver owing to precise control over microenvironmental parameters, including fluid flow, nutrient gradients, and mechanical cues. However, MPS are difficult to scale up and have not been developed to reproduce a MASH phenotype.

In comparison, herein we describe our bioprinted liver tissues for MASH, which have many advantages. Bioprinting enables the precise spatial control of the bioink and the deposition of the liver cells [27]. Our current 3D bioprinted liver tissues are comprised of major primary liver cells including hepatocytes, endothelial cells, stellates and Kupffer cells, mixed in physiologically relevant ratios. These cells are then bioprinted in precise pre-defined liver specific geometries to recapitulate complexity of the hepatic microenvironment. This mimicking of tissue architecture enables more accurate representation of cellular interactions, spatial organization, and microenvironmental cues, crucial for MASH pathogenesis. The liver tissues developed using this method maintain differentiation and metabolism over several weeks. In this paper, we demonstrated that upon treatment with a MASH induction cocktail, these liver tissues respond appropriately by developing steatosis and fibrosis. We were further able to show that the tissue response varied in accordance with the complexity of the MASH induction cocktail.

The bioprinted liver tissues are also of sufficient size to perform the same histological evaluation as a liver biopsy from a patient with MASH. This is a critical differentiating factor as the current FDA evaluation of drugs to treat MASH requires histological assessment of a liver biopsy. Further, it has been well accepted that the fibrosis stage and not steatosis or hepatocyte injury, correlates with mortality in patients with MASH. Histological assessment of the bioprinted tissues subjected to various MASH induction protocols demonstrated the presence of key hallmarks of MASH including ballooned hepatocytes, steatosis as well as fibrosis. Furthermore, donor specific differences in pathology could be visualized using this method, highlighting the benefit of this complex 3D MASH tissue model.

A major challenge for drug companies is predicting how well drugs that were effective in rodent models translate to clinical success. To date, nearly all drugs that have proven efficacious and safe in 2D cell cultures and MASH rodent models have failed to some degree to demonstrate similar efficacy or safety in MASH patients. Therefore, there is a critical need for more predictive multicellular human 3D in vitro MASH models to assess drug efficacy and safety, ultimately reducing the cost of failed drug testing in MASH clinical trials. To evaluate the efficacy of drugs targeting MASH phenotypes in 3D bioprinted liver tissues, we dosed our tissues with GS-0976, that has previously shown [28] to reduce steatosis in MASH patients. Tissues treated with this compound resulted in reduced steatosis compared to vehicle controls.

Transcriptional analysis by bulk RNA-seq provided new insights into the healthy and MASH bioprinted liver tissues. This analysis demonstrated the stability of hepatocyte gene expression in the healthy liver tissues as we had previously reported and in the MASH liver tissues. This contrasts with 2D cultures, where the transcriptome rapidly loses the expression of hepatocyte specific genes. The transcriptome of the MASH tissues differed significantly from the healthy livers and more closely resembled the transcriptome of biopsies of MASH livers from patients. Pathway analysis of the MASH bioprinted liver tissues identified the extracellular matrix and cirrhosis pathways as being significantly enriched. This result together with the histological analysis supported our claim that fibrosis, the most critical readout in MASH, can be effectively studied and monitored in our bioprinted liver tissues.

Despite the abovementioned benefits of our system, we recognize the shortcomings with the current format of these bioprinted liver tissues. Current bioprinted liver tissues do not reproduce the vasculature or biliary system of the liver [4]. Additionally, the liver tissues do not receive the physiological perfusion as in the MPS. Although, a key component of MASH is the recruitment of inflammatory/immune cells, our liver tissues are bioprinted with a fixed number of immune cells (mainly Kupffer cells) from normal human livers. In our next generation of liver tissues, we propose to bioprint inflammatory/immune cells from MASH livers to reflect the constituents of a diseased liver and to develop a perfusion system.

This work demonstrates that key features of MASH—steatosis, inflammation, hepatocyte injury, and fibrosis—can be effectively recapitulated in vitro using a 3D liver bioprinting approach combined with nutrient overload. Given that MAFLD/MASH is a silent disease, often diagnosed and sampled in patients only at advanced stages, this model presents an opportunity to expand our understanding of disease progression, identify early markers, and evaluate the efficacy and safety of therapeutic candidates within a human-relevant system.

## Supporting information

S1 Fig Bioprinted 3D tissues treated with MASH induction media consisting of sugars and fatty acids result in steatosis as seen in the image on the right. M–putative macrovesicular steatosis, m–putative microvesicular steatosis, B–putative ballooning hepatocytes.
(TIF)

S2 Fig Bioprinted 3D tissues treated with MASH induction media consisting of sugars and fatty acids result in statistically significant increase in inflammatory cytokines. (TIF)

S3 Fig Trichrome staining of sections of tissues treated with G/F/PA for 21 days show collagen deposition in cobalt blue, putative macrovesicular (M) and microvesicular (m) steatosis and putative ballooning hepatocytes (B). npc, non-parenchymal cells.
(TIF)

S4 Fig α-Smooth muscle actin (SMA) staining of sections of tissues treated with G/F/PA for 21 days shows increased hepatic stellate cell activation throughout the tissue compared to untreated control tissues. (TIF)
