# Supplementary figures and images for: Induction of MASH in three-dimensional bioprinted human liver tissue
Source: PLoS One. 2024 Dec 30;19(12):e0312615. doi: 10.1371/journal.pone.0312615 (PMC11684678; doi:10.1371/journal.pone.0312615)

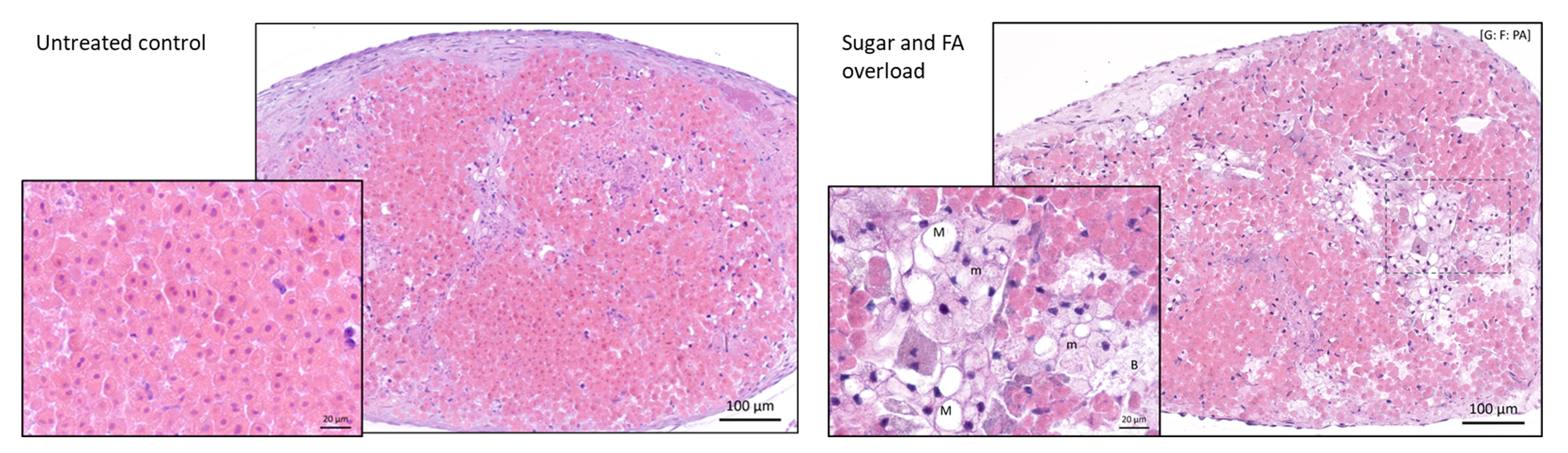

Supplement: S1 Fig — M–putative macrovesicular steatosis, m–putative microvesicular steatosis, B–putative ballooning hepatocytes. (TIF) [file pone.0312615.s001.tif]

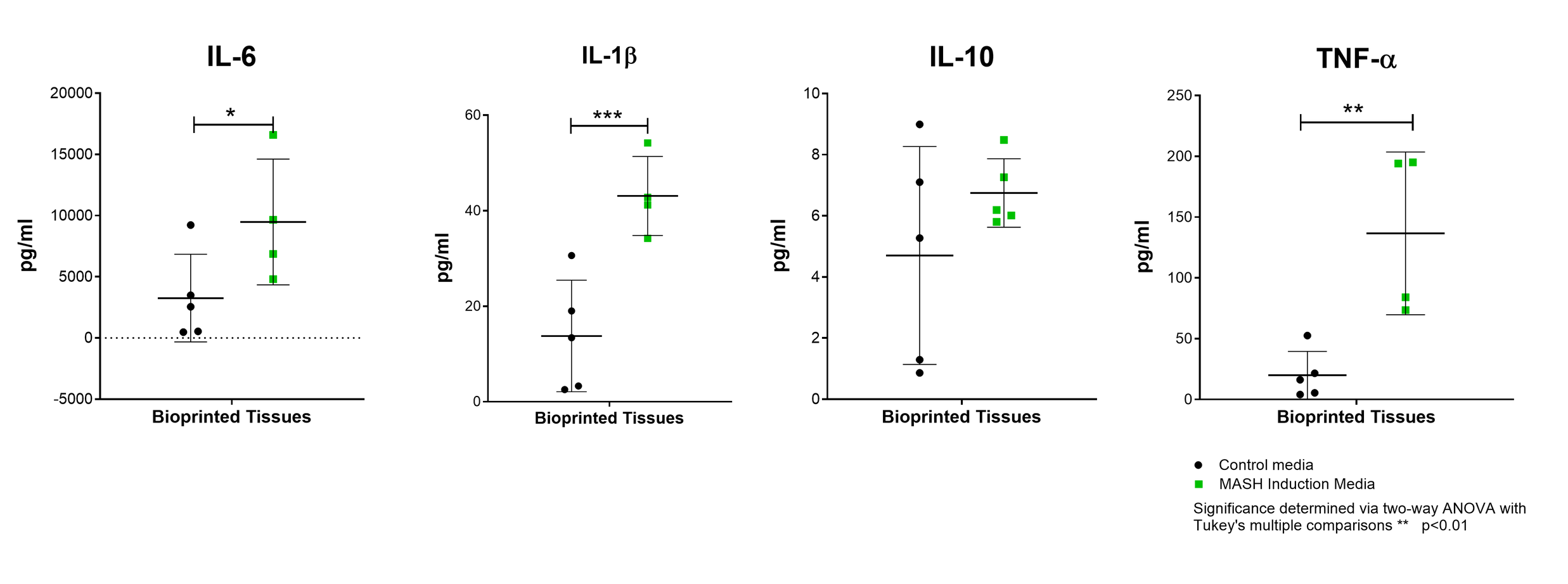

Supplement: S2 Fig — (TIF) [file pone.0312615.s002.tif]

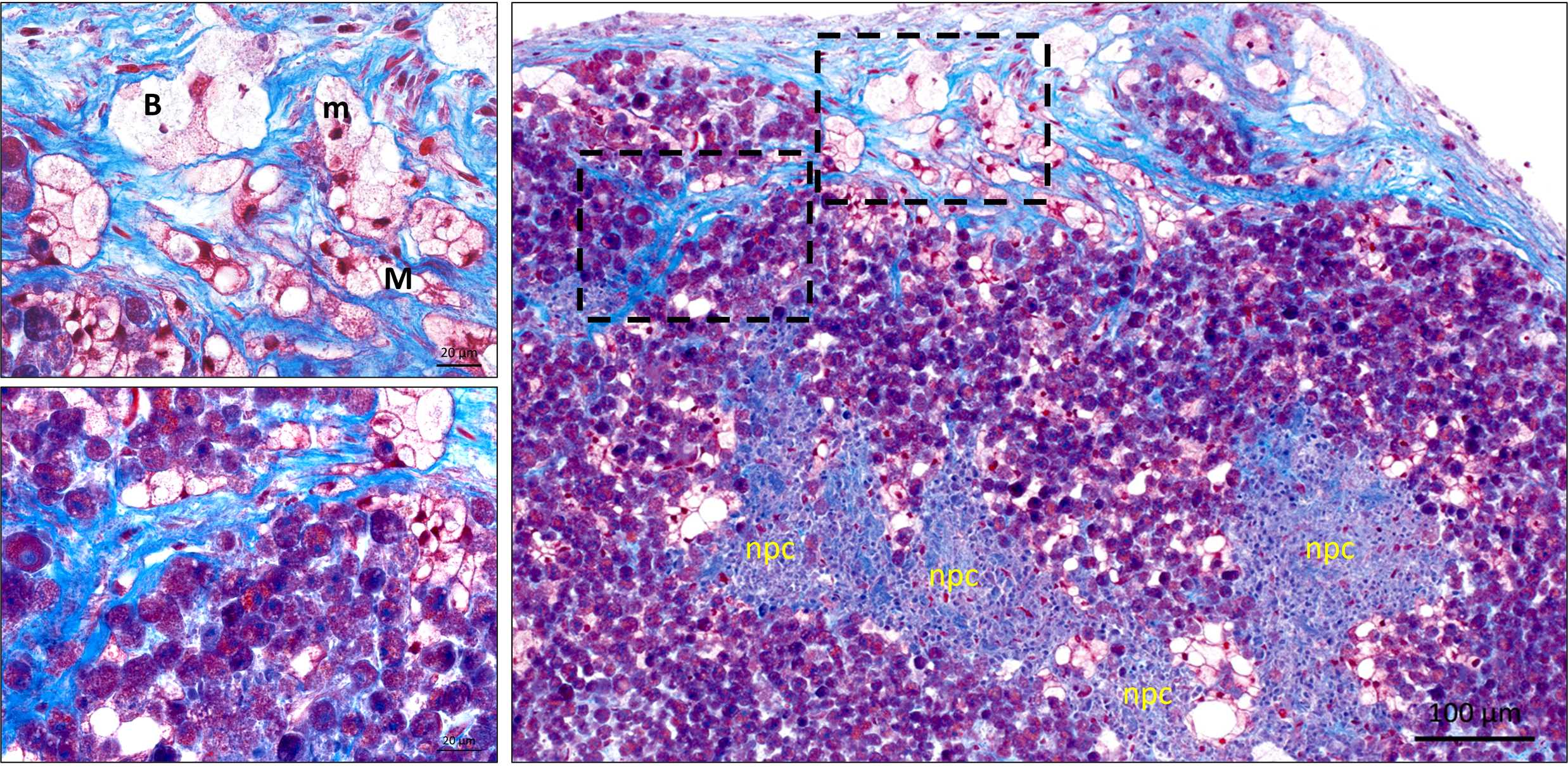

Supplement: S3 Fig — npc, non-parenchymal cells. (TIF) [file pone.0312615.s003.tif]

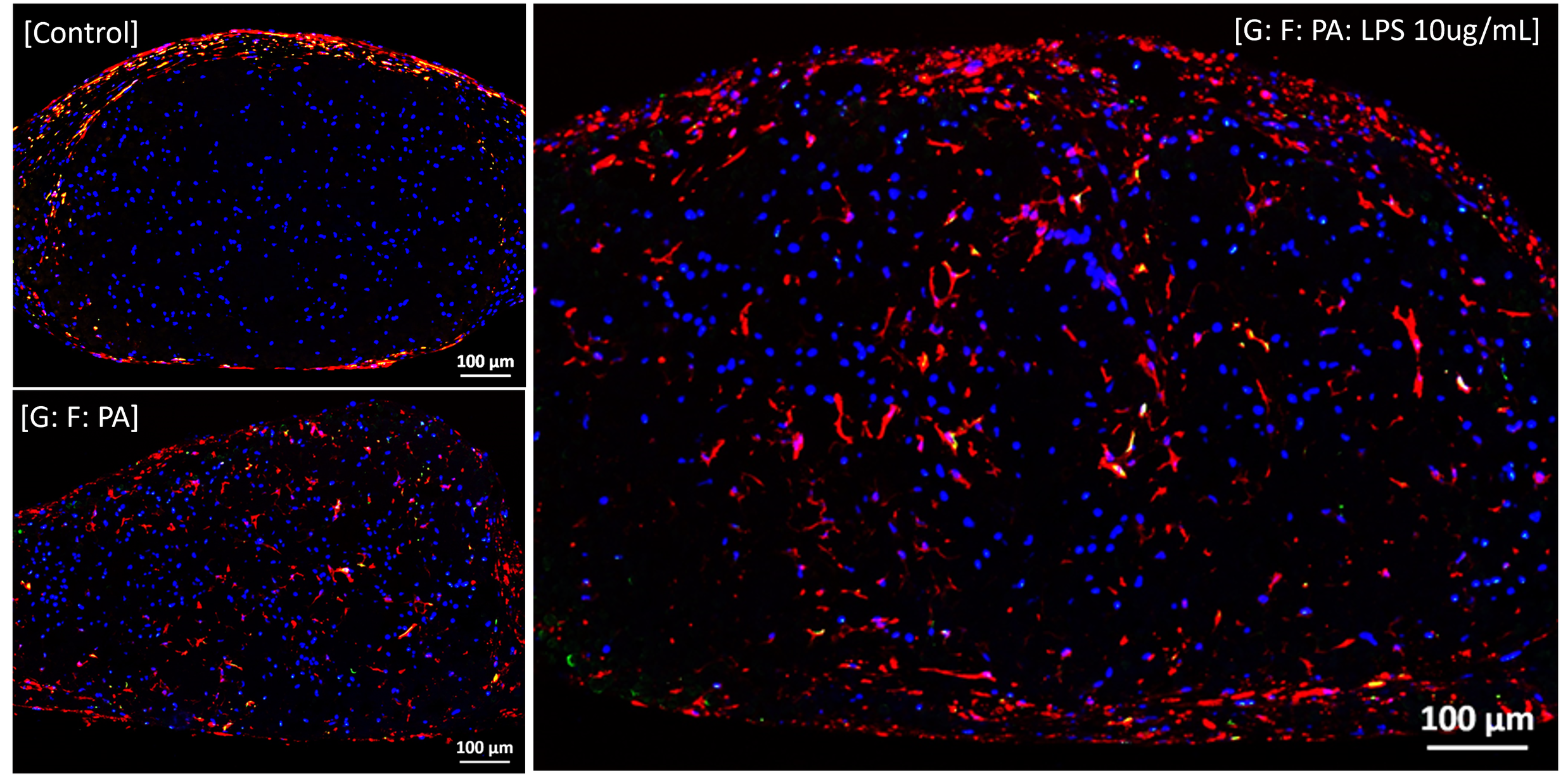

Supplement: S4 Fig — (TIF) [file pone.0312615.s004.tif]
